# Disparities in Cessation Behaviors Between Hispanic and Non-Hispanic White Adult Cigarette Smokers in the United States, 2000–2015

**DOI:** 10.5888/pcd17.190279

**Published:** 2020-01-30

**Authors:** Stephen Babb, Ann Malarcher, Kat Asman, Michelle Johns, Ralph Caraballo, Brenna VanFrank, Bridgette Garrett

**Affiliations:** 1 Office on Smoking and Health, Centers for Disease Control and Prevention, Atlanta, Georgia; 2 McKing Consulting Corporation, Fairfax, Virginia; 3 RTI International, Atlanta, Georgia

## Abstract

**Introduction:**

Hispanic adults make up a growing share of US adult smokers, and smoking is a major preventable cause of disease and death among Hispanic adults. No previous study has compared trends in smoking cessation behaviors among Hispanic adults and non-Hispanic white adults over time. We examined trends in cessation behaviors among Hispanic and non-Hispanic white adult cigarette smokers during 2000–2015.

**Methods:**

Using self-reported data from the National Health Interview Survey, we compared trends in quit attempts, receipt of advice to quit from a health professional, and use of cessation treatment (counseling and/or medication) among Hispanic and non-Hispanic white adult smokers. We also assessed these behaviors among 4 Hispanic subgroups. We conducted analyses in 2018–2019.

**Results:**

Past-year quit attempts increased during 2000–2015 among both non-Hispanic white and Hispanic smokers, with no significant differences between these groups. Receiving advice to quit increased significantly among non-Hispanic white adults but did not increase significantly among Hispanic adults. Cessation treatment use increased among both non-Hispanic white and Hispanic adults. Throughout 2000–2015, the prevalence of receiving advice to quit and using cessation treatments was lower among Hispanic adults than non-Hispanic white adults. In 2015, a higher proportion of Hispanic than non-Hispanic white smokers visited a health care provider without receiving advice to quit.

**Conclusion:**

Hispanic adult smokers are less likely to receive advice to quit and to use proven cessation treatments than non-Hispanic white smokers, and this pattern persisted over time. Culturally competent educational initiatives directed at both providers and Hispanic communities could help eliminate this marked and persistent disparity.

**Summary**

**What is already known on this topic?**

Several studies have reported that Hispanic smokers are less likely than non-Hispanic white smokers to receive advice to quit and to use cessation treatments. However, no previous study has explored how trends in these cessation behaviors compare among Hispanic and non-Hispanic white adults over time.

**What is added by this report?**

Throughout 2000–2015, the prevalence of receiving advice to quit and using cessation treatments was lower among Hispanic adults than non-Hispanic white adults. In 2015, a higher proportion of Hispanic than non-Hispanic white smokers visited a health care provider without receiving advice to quit.

**What are the implications for public health practice?**

Hispanic adult smokers are less likely to receive advice to quit and to use proven cessation treatments than non-Hispanic white adult smokers, and this pattern has persisted over time. Culturally competent educational initiatives directed at both providers and Hispanic communities could help eliminate this marked and persistent disparity.

## Introduction

In 2018, more than 3.9 million Hispanic adults in the United States reported current cigarette smoking, comprising about 11% of the 34.2 million US adult smokers, up from about 8% in 2000 (1). In 2018, Hispanic adults had a lower smoking prevalence (9.8%) than non-Hispanic white adults (15.0%) and US adults overall (13.7%) (2); however, smoking prevalence varies among Hispanic subgroups (3). Cigarette smoking is a major preventable cause of disease and death among Hispanic adults in the United States (4).

Smokers who receive advice to quit from a health professional and use individual, group, and telephone cessation counseling and 7 US Food and Drug Administration (FDA)-approved cessation medications are more likely to quit than those who do not receive advice or use counseling or medications (5,6). Clinical cessation guidelines recommend that health care providers consistently advise patients who use tobacco to quit and offer them cessation treatments (5,6). Disparities in receiving advice to quit and using proven cessation treatments can contribute to disparities in smoking cessation (5,7).

Several studies reported that Hispanic smokers are less likely than non-Hispanic white smokers to be screened for tobacco use, receive advice to quit, and use cessation treatments (7–17). Previous studies examined prevalence and trends in receiving advice to quit and using cessation treatments among the overall population of US adult smokers (7,14,18,19). However, no previous study explored how these trends compare among Hispanic and non-Hispanic white adults.

The primary goal of this study was to examine trends during 2000–2015 in the prevalence of quit attempts, receiving advice to quit, and using cessation treatments among Hispanic and non-Hispanic white adult cigarette smokers, as well as among Hispanic subgroups, to determine whether comparable progress in these cessation behaviors occurred across these groups during this period. In addition, for 2015, we examined 1) demographic correlates of current cigarette smoking among Hispanic adults overall, Hispanic subgroups, and non-Hispanic white adults, 2) the association of receiving advice to quit with making a past-year quit attempt and treatment use, and 3) demographic correlates of making a past-year quit attempt, receiving advice to quit, and treatment use. Our findings can inform efforts to address disparities in smoking cessation behaviors among Hispanic adults.

## Methods

Data came from the National Health Interview Survey (NHIS), an annual, nationally representative, in-person household survey of adults (aged ≥18) in the noninstitutionalized US civilian population. The NHIS sample adult core questionnaire is administered to a randomly selected adult in each sampled family and includes questions on cigarette smoking, quit attempts, recent successful smoking cessation, race/ethnicity, and other demographic and health-related characteristics. In 2000, 2005, 2010, and 2015, NHIS respondents were administered a supplemental questionnaire that contained questions on receiving advice to quit smoking and using cessation treatments. This supplement was last administered in 2015; as a result, this was the last time these questions were asked. NHIS adult core sample sizes and final response rates for 2000, 2005, 2010, and 2015 ranged from 27,157 to 33,672 and 55.2% to 72.1% (20).

### Measures

**Race/ethnicity.** We restricted this analysis to Hispanic adults and non-Hispanic white adults. Race/ethnicity was determined by self-report. For race, respondents were asked, “What race or races do you consider yourself to be?” To identify Hispanic ethnicity, respondents were asked, “Do you consider yourself Hispanic or Latino?” Those who answered affirmatively were also asked, “Please give me the number of the group that represents your Hispanic origin or ancestry.” Respondents could select up to 5 groups from among 8 options (Mexican, Mexican American, Puerto Rican, Cuban/Cuban American, Dominican [Republic], Central or South American, other Latin American, or other Hispanic/Latino/Spanish).

We compared Hispanic adults overall (answered yes to Hispanic ethnicity) with non-Hispanic white adults (answered “white” to the race question and no to Hispanic ethnicity). We also examined the following Hispanic subgroups: Mexican, Mexican American, Puerto Rican, and other Hispanic. The “other Hispanic” category includes Hispanic persons who indicated that their origin/ancestry was Cuban/Cuban American, Dominican [Republic], Central or South American, other Latin American, or other Hispanic/Latino/Spanish, and those who belonged to more than 1 subgroup. The “other Hispanic” category was created because small numbers prevented the generation of reliable estimates for the 6 groups included in this category.

**Cigarette smoking.** Current smokers were those who had smoked 100 or more cigarettes during their lifetime and, at the time of the interview, reported smoking every day or some days (7). Former smokers were those who reported smoking 100 or more cigarettes during their lifetime but currently did not smoke (7). We also assessed frequency and amount of cigarette smoking.

**Quit attempts.** Those attempting to quit were defined as 1) current smokers who reported stopping smoking for >1 day during the 12 months before the interview because they were trying to quit smoking and 2) former smokers who had quit in the past year (7,20).

**Advice to quit.** Current cigarette smokers and former smokers who had quit in the past year were asked whether they had received advice to quit smoking (or use of other tobacco products) from a health professional (limited to those who had seen a health professional in the past year).

**Cessation treatment use.** Separate questions were asked to assess use of cessation counseling and cessation medications. Response options varied by year. Response options for 2010 and 2015 included one-on-one counseling; a stop smoking clinic, class, or support group; a telephone help line or quitline; the nicotine patch; nicotine gum or lozenge; nicotine nasal spray or inhaler; varenicline; and bupropion. Response options for 2005 included a nicotine tablet and did not include varenicline (which was not approved by the FDA until 2006). Response options for 2000 included a stop smoking program and did not include a stop smoking class or support group, the nicotine lozenge (which was not approved by the FDA until 2002), and varenicline. Responses to these questions were used to assess treatments used in the past year by current cigarette smokers who had tried to quit in the past year and cessation treatments used when stopping smoking completely among former cigarette smokers who reported quitting within the past 2 years.

**Demographic characteristics.** We assessed the following demographic characteristics: sex, age, education, US region of residence, birthplace (born in United States or US territory or not), and health insurance coverage. Data were also collected on the language the survey was conducted in and self-rated English-speaking ability.

### Analysis

We conducted analyses by using SAS-callable SUDAAN version 11.0 (SAS Institute Inc). Data were adjusted for differences in probability of selection and nonresponse and weighted to provide national estimates. We used logistic regression models controlling for age and sex to analyze linear and quadratic time trends for each of the 3 dichotomous cessation measures (made a past-year quit attempt [yes or no], received advice to quit from a health professional [yes or no], and used cessation treatment [yes or no]). We used a Wald statistic to determine significance of the quadratic and linear β coefficients. We considered results significant at *P* < .05. If the quadratic term was not significant, we removed it from the model and assessed the significance of the linear time variable. We conducted analyses in 2018 and 2019.

We assessed demographic and cessation characteristics of Mexican, Mexican American, Puerto Rican, other Hispanic, all Hispanic, and non-Hispanic white current smokers in 2015. For 2015, we used multivariate logistic regression models for each cessation measure (past-year quit attempt, receipt of advice to quit, and treatment use) controlling for age, sex, education, region of residence, and health insurance to estimate odds ratios and 95% confidence intervals (CIs) for all Hispanic adults, the 4 Hispanic subgroups, and non-Hispanic white adults.

## Results

In 2015 overall, the prevalence of cigarette smoking was lower among Hispanic adults (10.1%) than among non-Hispanic white adults (16.6%); however, the prevalence of cigarette smoking among Puerto Rican adults (16.1%) and Mexican American adults (13.3%) was similar to the prevalence among non-Hispanic white adults (Table 1). A higher proportion of Hispanic smokers than non-Hispanic white smokers reported smoking some days and smoking 1 to 4 cigarettes per day. The prevalence of e-cigarette use was higher among non-Hispanic white cigarette smokers than Hispanic cigarette smokers. The proportion of current smokers who were male, were aged 18 to 24 years, had no high school diploma, resided in the West, and were enrolled in Medicaid or uninsured was higher among Hispanic adults than among non-Hispanic white adults. The proportion of current smokers whose survey was conducted in English, who said they speak English well or very well, and who were born in the United States or a US territory was lower among Hispanic adults than among non-Hispanic white adults.

**Table 1:** Prevalence of Cigarette Smoking Among Adults and Characteristics of Adult Current Cigarette Smokers,^a^ by Race/Ethnicity, National Health Interview Survey, United States, 2015^b^

Characteristic	Mexican	Mexican- American	Puerto Rican	Other Hispanic	Overall Hispanic	Non-Hispanic White
**Prevalence of cigarette smoking**	8.6 (7.3–10.2)	13.3 (11.1–16.0)	16.1 (12.7–20.1)	7.3 (6.0–8.8)	10.1 (9.1–11.1)	16.6 (15.8–17.4)
**Unweighted no. of cigarette smokers**	208	182	112	151	653	3,612
**Weighted no. of cigarette smokers, in millions**	1.2	1.1	0.6	0.8	3.8	26.0
**Male sex**	67.4 (59.2–74.6)	67.6 (58.0–76.0)	53.7 (41.9–65.2)	64.1 (54.1–73.0)	64.6 (59.7–69.2)	50.5 (48.1–52.9)
**Age group, y**						
18–24	10.3 (6.1–16.8)	21.5 (13.9–31.9)	—c	13.2 (7.4–22.6)	14.8 (11.3–19.2)	9.6 (8.1–11.2)
25–44	47.0 (39.1–55.1)	45.3 (35.2–55.8)	40.4 (30.6–51.0)	49.8 (39.2–60.4)	46.0 (40.9–51.2)	38.8 (36.3–41.3)
≥45	42.7 (34.3–51.6)	33.2 (25.1–42.4)	45.8 (34.9–57.1)	37.0 (27.9–47.2)	39.1 (34.2–44.3)	51.6 (49.1–54.2)
**Education^d^**						
No high school diploma (≤12 y)	55.6 (46.0–64.9)	30.4 (21.9–40.6)	33.0 (23.8–43.7)	36.1 (24.9–49.0)	40.7 (35.6–46.0)	16.4 (14.7–18.2)
High school diploma or GED	29.5 (21.3–39.4)	22.6 (15.0–32.6)	21.9 (12.9–34.5)	26.3 (17.8–37.1)	25.7 (21.1–30.9)	35.5 (33.3–37.7)
>High school diploma	14.8 (10.1–21.2)	46.9 (36.6–57.5)	45.2 (32.7–58.3)	37.6 (28.1–48.2)	33.6 (29.0–38.7)	48.1 (45.8–50.4)
**Percentage living below federal poverty level**	25.4 (18.1–34.3)	25.0 (17.9–33.9)	21.9 (13.9–32.7)	22.4 (15.2–31.6)	24.1 (20.0–28.7)	18.3 (16.6–20.0)
**Region^e^**						
Northeast	—c	—c	51.2 (39.1–63.2)	21.3 (14.4–30.4)	13.5 (10.4–17.4)	16.5 (14.5–18.7)
Midwest	12.7 (8.0–19.8)	13.4 (7.8–22.0)	—c	—c	11.7 (8.7–15.6)	32.1 (29.4–34.9)
South	39.0 (30.6–48.1)	41.6 (31.7–52.2)	36.3 (25.5–48.7)	41.4 (31.2–52.4)	39.8 (34.4–45.5)	34.6 (32.3–37.1)
West	46.8 (38.4–55.3)	43.7 (32.8–55.3)	—c	27.2 (18.4–38.2)	34.9 (29.4–40.8)	16.7 (15.0–18.6)
**Health insurance coverage**						
Private	29.0 (21.9–37.3)	41.9 (32.4–52.0)	30.3 (20.6–42.2)	40.0 (30.3–50.4)	35.4 (30.4–40.8)	52.8 (50.3–55.2)
Medicaid, including those eligible for Medicaid and Medicare	20.2 (13.4–29.4)	22.4 (16.5–29.6)	43.4 (32.7–54.9)	22.6 (14.2–34.1)	25.1 (20.9–29.8)	18.8 (17.0–20.8)
Other	10.0 (6.4–15.1)	—c	—c	—c	7.6 (5.5–10.4)	11.1 (9.8–12.5)
Uninsured	40.8 (32.6–49.5)	30.0 (20.0–39.5)	15.0 (8.8–24.5)	30.7 (21.5–41.9)	31.3 (26.6–36.4)	16.5 (14.7–18.4)
**English language proficiency**						
Completed survey in English language	45.7 (37.5–54.3)	86.2 (77.4–91.9)	81.2 (70.5–88.7)	63.8 (53.2–73.1)	67.4 (62.3–72.1)	99.5 (99.1–99.7)
Self-rated their English-speaking ability as speaking well or very well	52.4 (44.7–60.0)	95.2 (89.5–97.8)	90.4 (83.9–94.5)	68.4 (57.4–77.7)	74.7 (70.6–78.5)	99.6 (99.4–99.8)
**Born in the United States or US territory**	23.6 (17.2–31.4)	91.6 (84.7–95.6)	97.6 (91.2–99.4)	38.0 (28.3–48.9)	59.0 (54.0–63.7)	96.5 (95.4–97.3)
**Cigarette smoking frequency and amount**						
Someday smokers	49.6 (41.3–57.9)	47.8 (39.7–56.1)	31.5 (20.7–44.7)	44.8 (34.2–55.8)	45.2 (40.4–50.0)	20.4 (18.6–22.3)
1–4 Cigarettes daily	16.3 (11.4–22.7)	9.0 (5.4–14.5)	—c	10.2 (6.3–16.0)	11.8 (9.2–14.9)	4.6 (3.7–5.6)
5–14 Cigarettes daily	28.7 (21.5–37.1)	35.5 (27.1–45.0)	36.8 (26.7–48.1)	28.0 (20.0–37.8)	31.9 (27.4–36.7)	33.0 (30.9–35.2)
≥15 Cigarettes daily	5.4 (3.2–9.1)	7.7 (4.2–13.5)	21.9 (14.3–32.0)	17.1 (9.4–29.0)	11.2 (8.4–14.7)	42.0 (39.9–44.2)
**E-cigarette use**	—c	13.4 (8.6–20.4)	—c	—c	9.3 (6.8–12.4)	14.6 (13.0–16.4)

a Persons aged ≥18 years who reported smoking ≥100 cigarettes during their lifetime and who, at the time of the interview, reported smoking every day or some days.

b All values are percentage (95% confidence interval) unless otherwise indicated.

c Relative standard error >30% or denominator <50.

d Education is reported only for adults aged >25.

e Northeast: Connecticut, Maine, Massachusetts, New Hampshire, New Jersey, New York, Pennsylvania, Rhode Island, and Vermont. Midwest: Illinois, Indiana, Iowa, Kansas, Michigan, Minnesota, Missouri, Nebraska, North Dakota, Ohio, South Dakota, and Wisconsin. South: Alabama, Arkansas, Delaware, District of Columbia, Florida, Georgia, Kentucky, Louisiana, Maryland, Mississippi, North Carolina, Oklahoma, South Carolina, Tennessee, Texas, Virginia, and West Virginia. West: Alaska, Arizona, California, Colorado, Hawaii, Idaho, Montana, Nevada, New Mexico, Oregon, Utah, Washington, and Wyoming.

We observed differences in education, survey language, self-rated English-speaking ability, and birthplace among Hispanic subgroups (Table 1). For example, the proportion of adults who had more than 12 years of education was lower among Mexican adults than among the other 3 subgroups. The proportion of adults reporting Medicaid coverage was lower among Mexican and Mexican American adults than among Puerto Rican adults, and a higher proportion of Mexican adults than Puerto Rican adults was uninsured. The proportion of adults who reported smoking 15 or more cigarettes daily was lower among Mexican and Mexican American adults than among Puerto Rican adults.

### Quit attempts

**Figure 1:**
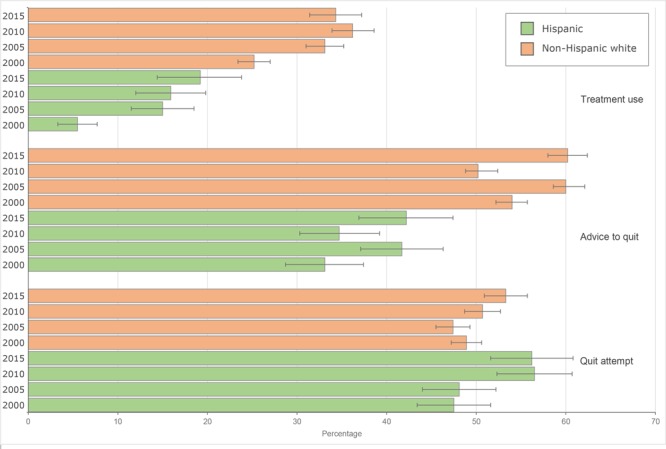
Prevalence of and change in past-year quit attempt, receiving a health professional’s advice to quit, and cessation treatment use among Hispanic and non-Hispanic white cigarette smokers aged ≥18 years, by year, National Health Interview Survey, United States, 2000–2015. Error bars indicate 95% confidence intervals.

Among Hispanic smokers, the prevalence of quit attempts increased from 47.5% in 2000 to 56.5% in 2010 (Figure 1). Among non-Hispanic white smokers, the prevalence of quit attempts increased from 48.9% in 2000 to 53.3% in 2015. Multivariate logistic regression models showed an increasing linear trend among Hispanic adults (*P* < .001) and a quadratic (ie, nonlinear) trend among non-Hispanic white adults (*P* = .03). Across all years, we observed no differences in the prevalence of quit attempts between Hispanic adults and non-Hispanic white adults.

**Table:** 

Year	Hispanic	Non-Hispanic White
**Quit attempt**		
2000	47.5 (43.4–51.6)	48.9 (47.2–50.6)
2005	48.1 (44.0–52.2)	47.4 (45.5–49.3)
2010	56.5 (52.3–60.7)	50.7 (48.7–52.7)
2015	56.2 (51.6–60.8)	53.3 (50.9–55.7)
**Advice to quit**		
2000	33.1 (28.8–37.5)	54.0 (52.3–55.8)
2005	41.7 (37.1–46.3)	60.0 (57.9–61.4)
2010	34.7 (30.2–39.1)	50.2 (48.0–52.3)
2015	42.2 (37.0–47.5)	60.2 (58.0–62.4)
**Treatment use**		
2000	5.5 (3.3–7.7)	25.2 (23.4–27.0)
2005	15.0 (11.5–18.5)	33.1 (31.0–35.2)
2010	15.9 (12.0–19.8)	36.2 (33.8–38.5)
2015	19.2 (14.6–24.0)	34.3 (31.4–37.2)

### Advice to quit

Among both Hispanic smokers and non-Hispanic white smokers who had visited a health professional in the past year, the prevalence of receiving advice to quit increased from 2000 to 2005, decreased from 2005 to 2010, and then increased from 2010 to 2015 (Figure 1). A multivariate logistic regression model confirmed a quadratic trend (*P* = .03) among non-Hispanic white adults. In contrast, among Hispanic adults, a similar model found no significant relationship between advice to quit and time. Throughout 2000–2015, the prevalence of receiving advice to quit was lower among Hispanic adults than among non-Hispanic white adults.

### Cessation treatment use

Among Hispanic adults, treatment use increased during 2000–2015 among current smokers who tried to quit in the past year and former smokers who quit during the past 2 years, with the largest increase from 2000 to 2005 (Figure 1). A large increase in treatment use from 2000 to 2005 occurred among non-Hispanic white smokers; however, their treatment use peaked in 2010. Logistic regression models indicated a quadratic trend among both Hispanic (*P* = .005) and non-Hispanic white (*P* < .001) smokers. When we examined each type of treatment separately, we found quadratic trends for all treatments among non-Hispanic white adults and Hispanic adults, except for use of nicotine patches and combination therapy among Hispanic adults, for which we found increasing linear trends (*P* = .002 and *P* = .01, respectively). Throughout 2000–2015, treatment use was lower among Hispanic than among non-Hispanic white smokers.

### Correlates of advice to quit, past-year quit attempt, and treatment use

After multivariate adjustment, Hispanic smokers had lower odds of receiving advice to quit (odds ratio [OR] = 0.51, 95% CI, 0.39–0.67) and of using cessation treatment (OR = 0.50, 95% CI, 0.34–0.71) than non-Hispanic white smokers in 2015. In similar models, the odds of receiving advice to quit were lower among Mexicans, Mexican Americans, and other Hispanic adults than among non-Hispanic white adults (range for ORs = 0.36–0.51) (Table 2). Mexicans had lower odds of using cessation treatment than non-Hispanic white adults (OR = 0.24, 95% CI, 0.12–0.48). In contrast with the other cessation behaviors, quit attempts did not vary significantly by race/ethnicity. Age, health insurance, and region of residence were also associated with these behaviors.

**Table 2:** Odds Ratios (95% Confidence Intervals) for Race/Ethnicity and Quit Attempt,^a^ Advice,^b^ and Treatment Use^c^ among Adults, National Health Interview Survey, United States, 2015

Characteristic	Quit Attempt^a^ (n = 4,784)	Advice to Quit^b^ (n = 3,848)	Treatment Use^c^ (n = 2,732)
**Race/ethnicity**			
Non-Hispanic white	1.0 [Reference]	1.0 [Reference]	1.0 [Reference]
Mexican	1.32 (0.94–1.86)	0.48 (0.32–0.73)	0.24 (0.12–0.48)
Mexican American	0.79 (0.53–1.20)	0.36 (0.22–0.59)	0.55 (0.28–1.11)
Puerto Rican	1.18 (0.71–1.95)	0.94 (0.53–1.64)	0.83 (0.41–1.67)
Other Hispanic	1.26 (0.80–1.99)	0.51 (0.30–0.86)	0.60 (0.31–1.14)
**Sex**			
Male	1.0 [Reference]	1.0 [Reference]	1.0 [Reference]
Female	1.04 (0.88–1.23)	1.07 (0.90–1.27)	1.04 (0.84–1.30)
**Age, y**			
18–24	1.0 [Reference]	1.0 [Reference]	1.0 [Reference]
25–44	0.68 (0.49–0.94)	1.19 (0.85–1.66)	2.66 (1.54–4.61)
≥45	0.43 (0.32–0.59)	1.93 (1.42–2.63)	4.57 (2.68–7.82)
**Education**			
No high school diploma (≤12 y)	1.0 [Reference]	1.0 [Reference]	1.0 [Reference]
High school diploma or GED	1.02 (0.80–1.31)	0.89 (0.69–1.15)	1.05 (0.72–1.54)
>High school diploma	1.19 (0.93–1.52)	0.91 (0.71–1.18)	1.08 (0.77–1.53)
**Health insurance coverage**			
Private insurance	1.0 [Reference]	1.0 [Reference]	1.0 [Reference]
Medicaid, including those eligible for Medicaid and Medicare	0.96 (0.77–1.19)	1.34 (1.04–1.73)	1.13 (0.84–1.54)
Other	0.83 (0.64–1.08)	1.42 (1.04–1.93)	0.79 (0.51–1.23)
Uninsured	0.81 (0.65–1.01)	0.85 (0.65–1.12)	0.75 (0.52–1.10)
**Region** d			
Northeast	1.0 [Reference]	1.0 [Reference]	1.0 [Reference]
Midwest	0.83 (0.62-1.11)	0.81 (0.60–1.09)	0.61 (0.41–0.90)
South	0.86 (0.67-1.10)	0.70 (0.53–0.92)	0.67 (0.46–0.97)
West	0.90 (0.67-1.21)	0.66 (0.49–0.89)	0.72 (0.48–1.10)

a Current smokers aged ≥18 who reported that they stopped smoking for >1 day in the past 12 months because they were trying to quit smoking and former smokers who quit in the past year.

b Received advice from a medical doctor, dentist, or other health professional to quit smoking or to quit using other kinds of tobacco, among current cigarette smokers and former cigarette smokers who quit in the past 12 months. The analysis was limited to current and former cigarette smokers who had seen a medical doctor or other health professional in the past year.

c Used one-on-one counseling; a stop smoking clinic, class, or support group; a telephone help line or quitline; nicotine patch, nicotine gum or lozenge, nicotine-containing nasal spray or inhaler, varenicline (US trade name, Chantix) and/or bupropion (including trade names Zyban and Wellbutrin) in the past year, among current smokers who tried to quit in the past year or used when stopping smoking among former smokers who quit in the past 2 years.

d Northeast: Connecticut, Maine, Massachusetts, New Hampshire, New Jersey, New York, Pennsylvania, Rhode Island, and Vermont. Midwest: Illinois, Indiana, Iowa, Kansas, Michigan, Minnesota, Missouri, Nebraska, North Dakota, Ohio, South Dakota, and Wisconsin. South: Alabama, Arkansas, Delaware, District of Columbia, Florida, Georgia, Kentucky, Louisiana, Maryland, Mississippi, North Carolina, Oklahoma, South Carolina, Tennessee, Texas, Virginia, and West Virginia. West: Alaska, Arizona, California, Colorado, Hawaii, Idaho, Montana, Nevada, New Mexico, Oregon, Utah, Washington, and Wyoming.

### Relationship between advice to quit, quit attempts, and treatment use

In examining the combined distribution of health care professional visits and advice to quit in 2015, the proportion of non-Hispanic white smokers who had visited a provider and received advice to quit was higher than the corresponding proportion of Hispanic smokers (54.7% vs 32.2%, respectively). A higher proportion of Hispanic than non-Hispanic white smokers either did not see a provider in the past year (27.7% vs 14.7%, respectively) or visited a provider but did not receive advice to quit (40.2% vs 30.5%, respectively). More than half (57.7%) of Puerto Rican smokers had visited a provider and received advice to quit, a prevalence closer to that of non-Hispanic white adults and higher than that of the other Hispanic subgroups.

**Figure 2:**
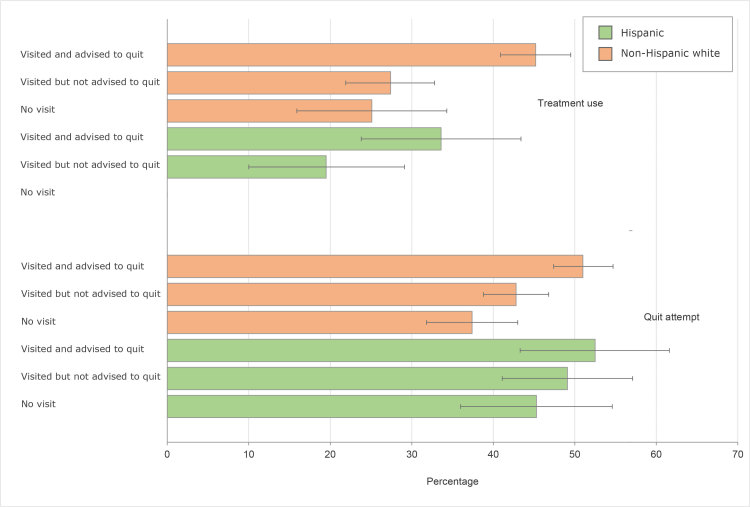
Prevalence of past-year quit attempt and cessation treatment use by provider advice among Hispanic current smokers and non-Hispanic white current smokers aged ≥18 years, National Health Interview Survey, United States, 2015. “No visit” indicates no visit to a health care provider in the past year. The value for prevalence of treatment use among Hispanic adults with no visit is not reported because of unstable estimates. Error bars indicate 95% confidence intervals.

Across all categories of visits and advice, the prevalence of quit attempts did not differ significantly between Hispanic adults and non-Hispanic white adults (Figure 2). Among non-Hispanic white adults, a past-year quit attempt was more frequent among those who received advice to quit (51.0%) than among those who saw a provider and did not receive advice (42.8%) and those who did not see a provider (37.4%). Similarly, among non-Hispanic white adults, use of cessation treatment was more frequent among those who received advice to quit (45.2%) than those who saw a provider and did not receive advice (27.4%) and those who did not see a provider (25.1%). In contrast, we observed no differences among Hispanic adults in quit attempts or use of cessation treatment between those receiving and not receiving advice to quit.

**Table:** 

Category	Hispanic	Non-Hispanic White
**Past-year quit attempt**		
No visit	45.3 (36.0–54.6)	37.4 (31.8–43.0)
Visited but no advice to quit	49.1 (41.1–57.1)	42.8 (38.8–46.8)
Visited and advised to quit	52.5 (43.3–61.6)	51.0 (47.4–54.7)
**Treatment use **		
No visit	Not reported because of unstable estimate	25.1 (15.9–34.3)
Visited but no advice to quit	19.5 (10.0–29.1)	27.4 (21.9–32.8)
Visited and advised to quit	33.6 (23.8–43.4)	45.2 (40.9–49.5)

## Discussion

During 2000–2015, past-year quit attempts and use of proven cessation treatments increased among both Hispanic and non-Hispanic white cigarette smokers. However, in 2015, although quit attempt prevalence did not differ between Hispanic adults and non-Hispanic white adults, Hispanic adults who tried to quit had 50% lower odds than non-Hispanic white adults of using evidence-based cessation treatment. Although receipt of health professionals’ advice to quit increased among both Hispanic adults and non-Hispanic white adults during 2000–2015, this increase was significant only among non-Hispanic white adults, and in 2015 Hispanic smokers who visited a provider in the past year had 51% lower odds of receiving advice to quit than non-Hispanic white adults. Disparities in advice to quit, compared with non-Hispanic white adults, were also observed among Hispanic subgroups, except Puerto Ricans. In contrast, only Mexican smokers had a significantly lower prevalence of cessation treatment use than non-Hispanic white smokers. In 2015, neither Hispanic nor non-Hispanic white adult cigarette smokers achieved Healthy People 2020 objective TU-4.1, which calls for 80% of adult smokers to make a past-year quit attempt (21), and levels of advice to quit and treatment use among both populations were suboptimal. However, in 2015 and throughout the study period, Hispanic smokers were significantly less likely than non-Hispanic white smokers to receive advice to quit and to use cessation treatments. These findings reinforce and extend the findings of previous studies that reported disparities between Hispanic and non-Hispanic white adults in tobacco use screening (8,9,16), advice to quit (7,9,10,13–15), and use of cessation treatments (7,9,11–14,16,17).

Cessation advice from a health professional increases the number of quit attempts, the use of cessation treatments, and successful cessation (5,19,22). We observed that more Hispanic than non-Hispanic white smokers had not seen a provider in the past year and therefore had no opportunity to receive such advice. Among Hispanic smokers, only about one-third (32.2%) visited a health professional and received advice to quit in the past year, compared with more than half (54.7%) of non-Hispanic white adults.

The disparities in receiving advice to quit among Hispanic smokers who saw a provider in the past year may be related to patient and provider characteristics (23–25). We observed that advice to quit varied by the patient’s age, health insurance coverage, and region of residence; however, these factors did not account for the observed disparity. Jamal and colleagues (8) found that adults who visited a primary care physician were more likely to be screened and to receive counseling than adults who visited a non-primary care physician. An age-adjusted analysis of the combined 2016 and 2017 NHIS found that, among adults aged 18 to 64, Hispanic adults (24%) were more likely to report having no usual source of medical care than non-Hispanic white adults (15%) (26). A lack of cultural competency or Spanish language skills among some providers may also contribute to the observed disparities (23–25). Receiving advice to quit was significantly associated with making a quit attempt among non-Hispanic white adults; however, although the pattern was similar, this association was not significant among Hispanic adults. The lack of significance of the relationship between advice to quit and quit attempts among Hispanic adults might be due to the smaller sample size for Hispanic adults in this study. Nevertheless, further research on the effectiveness of health professional advice to quit among Hispanic populations, including investigation of culturally tailored approaches, is warranted.

Lower prevalence of receiving cessation advice among Hispanic adults may have contributed to their lower rates of cessation treatment use compared with non-Hispanic white adults. Lower rates of insurance coverage, language barriers, or other access barriers encountered by Hispanic adults may also contribute to this disparity. Moreover, some Hispanic adults may have cultural beliefs that make them less likely to use cessation treatment, including beliefs that cessation medications are ineffective or dangerous, and that quitting is a matter of willpower (23–25,27,28). Finally, some Hispanic adults may distrust providers or the health care system, which could make them less receptive to advice to quit and less likely to use cessation treatment (24).

Targeted actions to address disparities in receiving advice to quit and using cessation treatment could be directed at both providers and Hispanic communities. Efforts to ensure that health care services and interventions are delivered in a culturally competent manner and that changes are made in health systems to ensure that providers engage every patient who uses tobacco at every visit could improve cessation outcomes, not only for Hispanic patients but for all patients (5,6,23–25,27,28). Culturally competent educational initiatives could also be conducted to increase knowledge among Hispanic adults about the availability and effectiveness of cessation treatments. Such initiatives could inform Hispanic adults about the proper use of cessation medications while putting their risks in context. Targeted educational initiatives could also increase awareness of free Spanish-language cessation resources such as 1–855-DÉJELO-YA (1-855–335–3569), a number operated by the National Cancer Institute that routes callers to Spanish-language services available from their state quitlines (29). These initiatives could appeal to Hispanic cultural values, such as love of family and respect for elders, while leveraging trusted advisors such as community leaders and *promotoras* (lay health workers), who can be effective channels for conveying health messages to some Hispanic populations (23–25). Finally, increased access to health care providers and to health insurance could, in combination with the actions described previously, increase receipt of advice to quit and use of cessation treatments among Hispanic adults (5).

This study has at least 3 limitations. First, smoking and cessation behaviors were self-reported, and might be subject to social desirability and recall biases. Self-reported smoking status has been shown to correlate with serum cotinine levels (30); however, few studies have examined the validity and recall of cessation measures. Second, low NHIS response rates might result in nonresponse bias. Finally, relatively small numbers of Hispanic respondents may have resulted in limited power, especially for subgroup analyses.

Marked disparities persist between Hispanic smokers and non-Hispanic white smokers in receiving advice to quit smoking and using proven cessation treatments. Eliminating these disparities will require concerted efforts. Because Hispanic adults make up a growing share of US adult smokers (1), and smoking is a major preventable cause of disease and death among Hispanic adults (4), these efforts can begin immediately, while research further explores the causes of these disparities.
